# Role of the Purinergic P2Y2 Receptor in Pulmonary Hypertension

**DOI:** 10.3390/ijerph182111009

**Published:** 2021-10-20

**Authors:** Mazen Shihan, Tatyana Novoyatleva, Thilo Lehmeyer, Akylbek Sydykov, Ralph T. Schermuly

**Affiliations:** Excellence Cluster Cardio-Pulmonary Institute (CPI), Universities of Giessen and Marburg Lung Center (UGMLC), German Center for Lung Research (DZL), Justus Liebig University of Giessen, Aulweg 130, 35392 Giessen, Germany; Tatyana.Novoyatleva@innere.med.uni-giessen.de (T.N.); Thilo.S.Lehmeyer@med.uni-giessen.de (T.L.); Akylbek.Sydykov@innere.med.uni-giessen.de (A.S.); Ralph.Schermuly@innere.med.uni-giessen.de (R.T.S.)

**Keywords:** pulmonary arterial hypertension, purinergic P2Y2 receptor, pharmacological approach

## Abstract

Pulmonary arterial hypertension (PAH), group 1 pulmonary hypertension (PH), is a fatal disease that is characterized by vasoconstriction, increased pressure in the pulmonary arteries, and right heart failure. PAH can be described by abnormal vascular remodeling, hyperproliferation in the vasculature, endothelial cell dysfunction, and vascular tone dysregulation. The disease pathomechanisms, however, are as yet not fully understood at the molecular level. Purinergic receptors P2Y within the G-protein-coupled receptor family play a major role in fluid shear stress transduction, proliferation, migration, and vascular tone regulation in systemic circulation, but less is known about their contribution in PAH. Hence, studies that focus on purinergic signaling are of great importance for the identification of new therapeutic targets in PAH. Interestingly, the role of P2Y2 receptors has not yet been sufficiently studied in PAH, whereas the relevance of other P2Ys as drug targets for PAH was shown using specific agonists or antagonists. In this review, we will shed light on P2Y receptors and focus more on the P2Y2 receptor as a potential novel player in PAH and as a new therapeutic target for disease management.

## 1. Introduction

Pulmonary hypertension (PH) represents a heterogeneous group of clinical entities, defined as a mean pulmonary artery pressure above 20 mmHg [1]. Pulmonary arterial hypertension (PAH) is a fatal disease, which belongs to group 1 PH and is defined as an elevation of mean pulmonary arterial pressure >20 mm Hg, with pulmonary arterial wedge pressure <15 mm Hg and pulmonary vascular resistance >3 Wood units at rest [2,3]. It is characterized by persistent vasoconstriction, thickening, and progressive obstructive remodeling of the pulmonary arteries. Hyperproliferation of pulmonary arterial smooth muscle cells (PASMCs), endothelial cells (PAECs), and microvascular endothelial cells plays an important role in pulmonary vascular remodeling [4].

The elevated pulmonary artery pressure in PAH is an outcome of pathological processes that involve the purinergic receptors P2Y, as a member of the G protein–coupled receptors family (GPCRs) [5,6,7,8,9,10,11,12,13]. Crucial mediators of vasodilation, such as nitric oxide (NO), are released from endothelial cells in response to elevated blood pressure and fluid shear stress (FSS) forces [14,15,16,17,18,19,20]. Hence, the endothelial dysfunction, in this context, can be defined as a decreased secretion of vasodilators and an increased secretion of vasoconstrictors, which contribute, along with other factors, to the vasoconstriction and pulmonary resistance increase [12,21,22,23,24]. Recent studies have demonstrated a purinergic receptor P2Y2 involvement in NO secretion, which results in systemic vasodilation, and suggested the P2Y2 receptor transmitted the FSS signaling in order to keep NO secretion at physiological levels [25].

**Figure 1:**
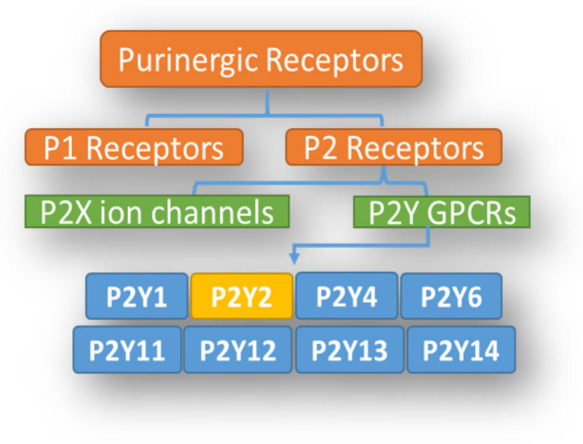
Purinergic receptor tree, and the classification of P2Y2: Adenosine receptors and ATP receptors (P1 and P2, respectively).

The lungs serve as a reservoir for numerous cell types, which express purinergic receptors. The purinergic receptors consist of two subfamilies: purinergic receptor 1 (P1), and purinergic receptor 2 (P2) (Figure 1). The P1 purinoreceptors are known as adenosine receptors and include four subtypes: A1R, A2AR, A2BR, and A3R [5]. The P2 purinoreceptors are categorized into two major sub-families: P2X, ligand-gated ionotropic membrane cation channels, and P2Y, metabotropic membrane bound, GPCRs (Figure 1) [26,27]. The P2Y receptors family comprises eight receptors (P2Y1, P2Y2, P2Y4, P2Y6, P2Y11, P2Y12, P2Y13, and P2Y14), with a high level of sequence similarity in transmembrane domains (especially P2Y1, P2Y2, P2Y4, and P2Y6), which does not easily allow synthetically developing specific ligands or pharmacologically discriminating one from other P2Y members, and forms an obstacle to selectively targeting one member of the P2Y family. Nevertheless, there are several structural homoplasies at the intracellular loops and the carboxy-terminus that make up the diversity and allow interactions with different G-proteins [28].

Alterations in purinergic signaling, as one of the potential mechanisms underlying PAH development, was recently reviewed by Cai et al. [5].The focus of this review article is the role of the P2Y2 receptor in PAH. The P2Y2 receptor is known to be associated with other diseases, such as dry eye syndrome [29,30], accommodative spasm [31], and cystic fibrosis [32,33]. This has stimulated many researchers to pharmacologically target P2Y2 to develop new treatment approaches for these diseases. Diquafosol tetrasodium, a uridine-5′-triphosphate (UTP) analogue and P2Y2 agonist that stimulates ophthalmic secretions has been approved as a therapeutic option for dry eye syndrome and accommodative spasm in some Asian countries [31,34]. Similarly, Denufosol (INS37217), another P2Y2 agonist, has reached phase III clinical trials to treat cystic fibrosis patients. Inhaled Denufosol restored sodium and chloride exchange and improved the airway clearance by enhancing ciliary beat frequency in patients with cystic fibrosis and normal to mildly impaired lung function [35,36,37]. Little is known, so far, about the potential role of P2Y2 receptors in PH, but this is still in the growth phase, and more information and new knowledge will, hopefully, emerge in the next few years.

## 2. P2Y2 Expression in Pulmonary Parenchyma, Vasculature, and Inflammatory Cells

P2Y2 has been demonstrated to be expressed on the protein level in primary rat type I alveolar epithelial cells (type I AECs) [38]. P2Y2 mRNA and protein expression were also noted, both in primary isolated rat type II AECs, and in immortalized tumor-derived AEC lines (L2, R3/1, RLE, and A549 cells) [39]. The epithelial expression of P2Y receptors in the alveolus is critically important for the surfactant secretion and the regulation of the alveolar surface liquid volume. P2Y2 activation in type II AECs in the alveolus occurs in response to stretch-induced adenosine-5′-triphosphate (ATP) release, which later, via subsequent activation of P2X4 receptors, results in surfactant release [40].

P2Y receptors were found in the pulmonary arteries of various species. Thus, mRNA expression of P2Y1, P2Y2, P2Y4, P2Y6, and P2Y12 receptors was demonstrated in rat pulmonary arteries [41,42,43]. Furthermore, mRNA expression of P2Y1, P2Y2, P2Y4, P2Y6, and P2Y12 was shown in mouse lung homogenates [44]. P2Y2 transcripts, concomitantly to the other members of the P2Y family, such as P2Y1 and P2Y4, were detected in the pulmonary arteries, peripheral lung tissues, and more specifically in PAECs of juvenile rabbits (Table 1) [45]. The P2Y receptors are broadly expressed in the endothelial cells of the pulmonary vasculature, suggesting their crucial importance for endothelial cell dysfunction, a pathophysiological process characterized by upregulation of adhesion molecules, increased chemokine secretion, leukocyte adherence, increased permeability, and vasoconstriction. The mRNA of P2Y2 was shown to be present in human microvascular endothelial cells from lung and PAECs [46,47,48]. Along with P2Y2, the expression of other P2Y receptors, such as P2Y1, P2Y6, and P2Y11 was detected in PAECs isolated from PAH patients and control human lungs [49,50,51].

There is accumulating evidence of the involvement of inflammation in the pathogenesis of PAH [56,57,58]. Pulmonary vascular lesions in PAH are associated with perivascular inflammation and inflammatory cell infiltration [56,59]. This pulmonary vascular inflammation in PAH has been shown to contribute to progressive pulmonary vascular remodeling [59]. In this regard, it is interesting to note that P2Y2 receptor expression was also revealed in the cells of the innate immune system, such as microphages and neutrophils. P2Y2 receptor transcripts were detected in human [60] and rat alveolar macrophages [61]. Furthermore, P2Y2 expression was also confirmed in human and rat neutrophils and eosinophils [62,63,64,65]. Macrophage-derived P2Y2 receptors sense the ATP and UTP released by apoptotic cells during immune responses to microbial infection. P2Y2 activation on alveolar macrophages results in an increase of intracellular Ca^2+^, which facilitates phagocytosis [40].

## 3. Deletion, Overexpression, and Disorders of P2Y2

### 3.1. Global P2Y2 Knockout

Compared to wild type (WT) mice, P2Y2 knockout mice (P2Y2-KO) showed no marked changes in body weight, glucose plasma concentrations, plasma osmolality, blood or urine pH, food and fluid intake, total fecal mass, or urinary fluid excretion, but P2Y2-KO mice exhibited lower plasma concentrations of aldosterone, renin, hematocrit, and K^+^ [66,67]. Global P2Y2 inactivation impaired the purinergic and Cl^-^ secretory responses in the airways (trachea), gallbladder, and intestines [55,68]. A comparison of P2Y2-KO to WT mice showed the most effects in trachea, and to lesser extent in the gallbladder and intestine [55,68,69]. The phenotype of P2Y2-KO mice suggests that the biological function of P2Y2 can be compensated by P2Y4 and P2Y6 receptors in other organs [55,68]. Further insights gained from these mice suggest that epithelial P2Y2 in trachea interacts with extracellular ATP to trigger Ca^2+^ mobilization, and, therefore, it is necessary for clearance responses, such as mucin, water secretion, and ciliary beat frequency [33,35,70,71].

In another study, double knockout (P2Y1/P2Y2) mice displayed 1.8-fold lower survival rates in comparison to WT mice, when exposed to an intra-tracheal instillation of Pseudomonas aeruginosa [72]. Thus, P2Y2 is suggested, together with P2Y1, to play a protective immunological role and have a pro-inflammatory cytokine response in bacterial infection [72], pneumonia virus infection [73], and to regulate VCAM1 in eosinophils during lung inflammation [74]. Neutrophils from P2Y2-KO mice exhibited loss of sensing, orientation, and chemotaxis, mediated by A3 and P2Y2 receptors [65,75]. P2Y2 also serves as a sensor for the ATP released by apoptotic cells to promote phagocytic clearance, which was decreased in P2Y2-KO mice. Thus, P2Y2 receptor is considered as a sensor for the critical nucleotides released by apoptotic cells as a ‘find-me’ object for phagocytosis [76].

Compared to WT mice, P2Y2-KO mice displayed a larger infarct size and worse heart function in a myocardial infarction model, and in vivo treatment with MRS2768, a P2Y2 specific agonist, protected the heart from ischemic damage [77,78] and prevented vascular calcification [79,80]. Mice lacking P2Y2 developed salt-resistant systemic hypertension [81] and impaired ATP- and ATPyS-evoked relaxation in aorta compared to WT mice, suggesting the importance of this receptor for vascular signaling [82].

### 3.2. Conditional (Inducible) Knockout of P2Y2

Conditional P2Y2 knockout (P2Y2-CKO) mice were generated by crossing floxed P2Y2 with endothelial Cre-Tie2 mice to specifically target endothelial P2Y2. Endothelial P2Y2-CKO mice exhibited increased vascular tone and elevated blood pressure [25]. Furthermore, endothelial P2Y2 was shown to be essential for mechanotransduction and FSS effects [25].

Endothelial P2Y2-CKO was suggested to promote plaque stability in atherosclerosis ApoE-KO mouse model through reduced macrophage infiltration and matrix metalloproteinase-2, which increases smooth muscle cell migration [83].

### 3.3. P2Y2 Overexpression

Transgenic rats overexpressing P2Y2 by implementing a lentiviral vector [84] exhibit dramatically accelerated neointimal hyperplasia when subjected to femoral artery injury, which gives clues about the inflammatory role of P2Y2 [85].

Another in vivo study showed that hypoxia-inducible factor-1α (Hif1α) activates transcriptional expression of P2Y2 in human primary renal tubular cells [86]. These findings were further confirmed in another study that showed that HIF-1α upregulated P2Y2 expression at mRNA and protein levels and, thereby, prolonged the viability of human hepatocellular carcinoma (HHC), whereas treatment of HCC with a selective P2Y2 antagonist MRS2312 led to a reduction in cell viability [87].

FSS was associated with an increment of P2Y2-mRNA levels in HUVECs after 6 h of exposure. In HUVECs transiently expressing the P2Y2 Arg-Gly-Glu mutant receptors, FFS effects altered cell-alignment, actin stress-fiber formation, phosphorylation of focal adhesion kinase, cofilin-1, and wound repair and healing [88,89].

Finally, P2Y2 mRNA has been shown to be significantly upregulated 2 days post spinal cord injury in rats, suggesting a P2Y2 responsiveness to altered conditions and mechanical injury [90].

## 4. P2Y2 Signaling

**Figure 2:**
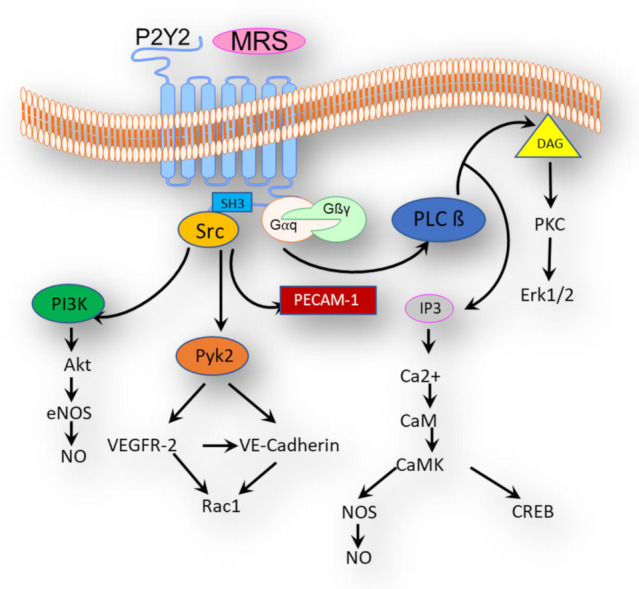
P2Y2 mediated signaling pathways that are generally expected in different cell types and tissues, including the pulmonary vasculature. Abbreviations: MRS = MRS2768; DAG = diacylglycerol; PKC = proteinkinase C; Erk1/2 = extracellular-signal regulated kinases; PLCβ = phospholipase C (β); IP3 = inositol trisphosphate; CaM = calmodulin; CaMK = Ca^2+^/calmodulin-dependent protein kinase; CREB = cAMP response element-binding protein; NOS = nitric oxide (NO) synthase; SH3 = SRC homology 3 domain; Gα, Gβ, and Gγ = G-protein subunits (α, β and γ); Src = proto-oncogene tyrosine-protein kinase; PECAM-1 = platelet endothelial cell adhesion molecule; Pyk2 = protein tyrosine kinase 2 beta; VEGFR-2 = vascular endothelial growth factor receptor 2; VE-cadherin = vascular endothelial cadherin; Rac1 = Ras-related C3 botulinum toxin substrate 1; PI3K = phosphoinositide 3-kinases; Akt = protein kinase B (PKB) [3,25,91,92,93,94,95,96,97,98,99,100,101].

P2Y2-mediated signaling pathways are summarized in Figure 2. P2Y2, like other GPCRs, is activated by extracellular stimuli. Conformational changes of P2Y2 receptors, therefore, induce intracellular signaling by activating heterotrimeric G-proteins, amongst other mechanisms. The activation of P2Y2 initiates a guanine nucleotide exchange factor (GEF), which leads to activation of subunit(s) of the coupled G-proteins. The activation/phosphorylation of one of the G-protein subunits (α, β, and γ) leads to its dissociation from the heterotrimeric complex, to start, in turn, a set of subsequent reactions and multiple effector proteins [3].

G-proteins comprise three main families: alpha (α), beta (β), and gamma (γ). Each family consists of subfamilies, e.g., the α subunit (16 subunits) forms four main subfamilies Gα_q_, Gα_s_, Gα_i/o_, and Gα_12/13_. Similarly, β and γ form two subfamilies, of 5 and 13 subunits, respectively. While β and γ have not been sufficiently studied regarding their interactions with P2Y2, other α subunits such as Gα_q_, Gα_o_, and Gα_12_, but not Gα_12/13_, are known to interact with P2Y2, due to their presence, distribution, and tissue specificity. Interestingly, researchers believe that G-proteins and GPCRs can elicit their actions and activate signaling pathways apart from each other; the so-called canonical and non-canonical signaling of GPCRs [102,103,104,105]. Furthermore, it has been shown in astrocytoma cells that, contrary to G_q_, coupling of P2Y2 to G_o_ and G_12_ requires an interaction with alpha v integrin [91,92].

Activation of Gα_q_ leads to catalyzation of phosphatidylinositol 4,5-bisphosphate by phospholipase C-β (PLCβ) into inositol trisphosphate (IP3) and diacylglycerol (DAG). Both IP3 and DAG activate the intracellular Ca^2+^ channels in the endoplasmic reticulum (ER) and Ca^2+^ ions released into the cytosol in pulmonary arterial vasa vasorum endothelial cells and pulmonary fibroblasts [8,55]. The binding of Ca^2+^ to calmodulin (CaM) activates calmodulin-dependent kinase, which is known to phosphorylate endothelial nitric oxide synthase (eNOS) to generate more NO. DAG, however, can activate the protein kinase C, which is also necessary for eNOS and extracellular signal-regulated kinases 1/2 (Erk1/2) phosphorylation [106].

Furthermore, P2Y2 activates growth factor receptors or receptor tyrosine kinases, as well as non-receptor tyrosine kinases, such as Src [107]. Src is transiently associated and activated by P2Y2 via SH3-binding domains in the C-terminal tail of G_q_-coupled GPCR [107]. Activation of Src, via tyrosine kinase proline-rich tyrosine kinase 2 (Pyk2), leads to phosphorylation of several growth factor receptors, including EGFR and VEGFR-2. The latter results in endothelial-leukocytes interactions and attachment of the leukocytes to the vessel wall [108].

Pyk2 plays a crucial role in the pathogenesis of PH. It is, however, important for hypoxia-induced proliferation and migration of human PASMCs. Furthermore, it is essential for reactive oxygen species (ROS) generation during hypoxic stress and the activation of Hif1α [109]. In addition, Src-phosphorylation leads to activation of serine/threonine-kinases such as ERK1/2, JNK, and p38, in addition to various transcription factors that are involved in inflammation, apoptosis, and cell differentiation (e.g., CREB, NF-kB, ELK1, c-FOS, c-JUN). Moreover, Src also activates the monomeric G-protein Rac1 and the platelet endothelial cell adhesion molecule-1 (PECAM-1), as well as phosphatidylinositol 3-kinase (PI3K), which phosphorylates protein kinase B (Akt), and which can also activate eNOS and enhance endothelial NO release [25,106].

Studies have shown P2Y2 to mediate phosphorylation of VE-cadherin via Src, Rac1, and VEGFR-2 [108,110]. The latter is essential for the endothelial regulation of angiogenesis, blood vessel permeability, and leukocyte trafficking [110].

In systemic circulation, Wang et al. demonstrated that G_q_/G_11_-coupled to P2Y2 activates a mechanosensory complex consisting of PECAM-1, VE-cadherin, and VEGFR-2 in response to FSS. Consequently, and in response to FSS, eNOS is activated in a Ca^2+^-calmodulin-dependent manner, but also in a Ca^2+^-calmodulin-independent manner through Akt in pulmonary artery adventitial vasa vasorum endothelial cells [8]. The generation of NO in the vessel wall leads to vasodilatation in smooth muscle cells [25].

Other studies also referred to the NO-independent-generation of Ca^2+^-calmodulin cascades [111]. They suggested an endothelial-derived hyperpolarization response via mechanisms involving potassium channels (KCa3.1) and connexin 37 (Cx37) [112]. Furthermore, P2Y2 signaling cascades were shown in small pulmonary veins, which were found to contract strongly upon P2Y2 activation in vascular smooth muscle cells as a result of IP3/DAG activation of Ca^2+^ channels in ER and cytosolic Ca^2+^ release [6].

In general, the activation of GPCRs can result in receptor desensitization, mediated by a family of GPCR kinases (GRK1-7), which supports the binding of β-arrestins to the receptor [113,114,115,116]. β-arrestins then inhibit the interaction between GPCRs and G-proteins (desensitization), promote internalization of the GPCRs into clathrin-coated pits (endocytosis), and interact, for example, with MAPK/Src signaling in rat aortic smooth muscle cells (ASMCs) [115]. The desensitization of GPCRs does not only lead to less activation by agonists, but also to their internalization [3,117]. Furthermore, GPCR desensitization is crucial for the sufficient activation of store-operated calcium release activated calcium channels, which leads to an influx of extracellular calcium [116]. In rat mesenteric arterial smooth muscle cells, β-arrestin 2 and GRK2 mediate the P2Y2 receptor desensitization [114,115,118,119]. Apart from GPCRs and G-proteins, canonical, and noncanonical signaling, β-arrestins have been suggested to be, at least partially, responsible for the P2Y2-mediated MAPKs and ERK1/2 activation, as reported in pulmonary artery adventitial vasa vasorum endothelial cells and ASMCs [8,113,114,115].

So far, no specific signaling cascades have been described for P2Y2 in PAECs or PASMCs, but various P2Y2 signaling cascades were linked to inflammatory responses and pathogenesis of atherosclerosis and other cardiovascular diseases. Thus, studies have suggested that activation of endothelial P2Y2 is involved in oxLDL-mediated inflammasome activation and subsequent IL-1β production, through the modulation of mtROS-mtDNA and TLR9-NFkB signaling pathways in human endothelial cells [120]. Other studies, however, showed that P2Y2 in vascular smooth muscle cells activates Nox1 via Rac, which leads to ROS generation and, thus, to oxidative stress, and that P2Y2 mediates proinflammatory signaling by monocyte chemo-attractant protein 1 formation, leading to an atherogenesis response [121].

Finally, activation of G_ο_ through P2Y2 is reported to mediate the activation of RhoA [91,108], whereas activation of Gα_12_ through P2Y2 is shown to activate Rac, RacGEF, and Vav2, which play a significant role in cell migration [91,110]. This suggests multiple signaling cascades and various responses that can be triggered by P2Y2, due to the broad distribution in organs and tissues [3]. Most of these signaling events have not yet been confirmed in the pulmonary vascular system and parenchyma, which prompts us towards more investigations, to clarify P2Y2 signaling in the pulmonary vasculature.

## 5. Physiological Function and Dysfunction of P2Y2

P2Y2 is associated with several diseases such as dry eye syndrome [29,30,122,123], accommodative spasm [123], and cystic fibrosis [32,33,35,36,124,125]. This illuminates the physiological importance of P2Y2 in different organs and biological systems, which can be summarized as follows:

### 5.1. Control of Vascular Tone (Vasorelaxation or Vasoconstriction)

Upregulation of vasoconstrictive mediators in PAECs and PASMCs, such as endothelin-1 and endothelin receptor A (ETA), which is accompanied by downregulation of vasodilatory mediators such as NO, prostacyclin (PGI2), endothelium-derived hyperpolarizing factor (EDHF), or prostaglandin receptors (DPs, EPs, FP, IP, or TP), is one example of the imbalance of GPCR-expression that contributes to the increased pulmonary vascular resistance in PAH patients [126]. Moreover, this imbalance in endothelial secretion systems is accompanied by disturbed G-protein expression in both PAECs and PASMCs [45,127,128]. Endothelial P2Y2 was shown to trigger vasodilatory function in response to increased FSS in systemic circulation [25]. In contrast, ATP and UTP were reported to play a vasoconstrictive role, probably via P2Y2, in intrapulmonary arteries isolated from normal and pulmonary hypertensive newborn piglets [12]. Mechanical injury, FSS, and hypoxia can also upregulate P2Y2, adding more complexity to the vascular control system [86,87,88,90,129,130,131,132]. It should be noted that ATP and ADP are non-selective P2Y2 receptor agonists, and therefore, more studies are required to investigate and clarify any vasodilatory or vasoconstrictive role of endothelial P2Y2 [133]. However, using PAECs- and PASMCs-cell specific P2Y2 knockout mice, and the application of the only available novel selective P2Y2 receptor agonist MRS2768, can provide more insights into the role of P2Y2 in pulmonary vascular regulation [134,135,136,137].

Endothelial P2Y2 coupled to Gα_q_/Gα_11_ is involved in systemic hypertension via transmission of FSS-induced phosphorylation of Akt, which, in turn, phosphorylates eNOS [25,138]. Under FSS forces, a variety of vasodilatory mediators, such as NO, PGI2, and EDHF, are released by endothelial cells in the systemic circulation [139,140,141,142,143,144]. NO produced by eNOS plays a potent role in flow-induced vasorelaxation [83,145]. Activation of P2Y2 results in decreased blood pressure through regulation of renal Na^+^, potassium channel, and connexin37 [112,146].

### 5.2. Mechanotransduction of FSS

**Figure 3:**
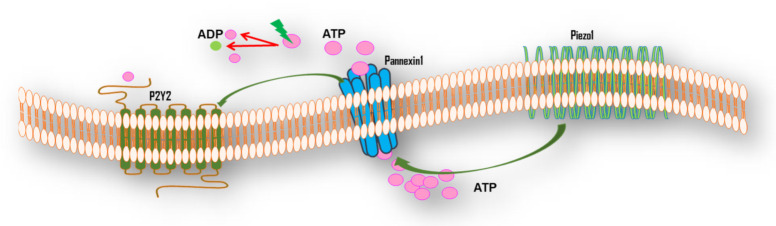
Endothelial P2Y2 interactions with Pannexin1 and Piezo1 under fluid shear stress conditions in systemic circulation. Fluid shear stress activates Piezo1, a mechanosensitive channel that mediates ATP release via pannexin1 channels. Ectonucleotidases hydrolyze the extracellular nucleotide ATP to ADP, AMP, and adenosine, which activate P2Y2 and other P2Y receptors, suggesting P2Y2 as being downstream of Piezo1 [99,145,151,152,153,154].

Similar to other cell types, endothelial cells release ATP via the pannexin1 channel in response to FSS, which activates the purinergic P2Y2 receptor (Figure 3) [145,147]. Furthermore, P2Y2 is a mechanosensitive channel, located downstream of Piezo1, suggesting a complicated signaling that ends in enhanced eNOS phosphorylation and NO release [99,145]. Hence, P2Y2 controls vascular tone and systematic blood pressure via NO, which, in turn, stimulates soluble guanylyl cyclase in vascular smooth muscle cells, resulting in increased cyclic GMP concentrations and vasodilatation [111,142,148,149,150]. In addition, under FSS conditions, there is P2Y2 upregulation [88,129,130,131,132].

### 5.3. Involvement in Phagocytic Clearance

Injury to pulmonary vascular cells is one of the mechanisms involved in PAH initiation and progression [155]. Apoptosis is an important regulator of tissue integrity and homeostasis, removing damaged or non-functional cells. However, it causes cellular damage [155]. There is evidence suggesting that the mismatch between apoptosis and apoptotic cell clearance underlies various pathologic conditions, including PAH [155,156].

The ATP release by different cell types, which is a part of the vascular tone regulation mechanisms, has another biological function. It participates in the phagocytosis of apoptotic cells and involves inflammatory cells such as monocytes, dendritic, macrophages, eosinophils, and neutrophils in the circulation system via inflammatory P2Y2 sensing [60,76,156,157]. Thus, ATP release from different apoptotic cells in pulmonary tissues, which is sensed by mast cells, eosinophils, dendritic cells, and other inflammatory cell types, suggests a significant inflammatory clearance role for ATP, UTP, ADP, and P2Y2 receptors in asthma, chronic obstructive pulmonary disease, cystic fibrosis, and other pulmonary diseases [30,32,33,35,37,70,73,76,125,128,158,159,160,161,162].

### 5.4. Proliferation/Anti-Apoptosis and Vascular Remodeling

Hyper-proliferation of PASMCs and PAECs results in lumen narrowing of the pulmonary arteries and increased pulmonary arterial pressure. The molecular mechanism involves a number of GPCRs, such as AT1, ETA, ETB, 5-HT1B, 5HT2A, 5HT2B, and PAR1-3, in addition to the G-proteins G_q_/G_11_ and G_12_/G_13_ [126]. P2Y2 mediated increased proliferation of breast cancer cells [163], human cancerous pancreatic duct epithelial cells [164], rabbit corneal endothelial cells [93], hepatocytes [165]. Furthermore, P2Y2 promoted vascular endothelial and smooth muscle cell proliferation [3,85,93,97]. The role of P2Y receptors in vascular homeostasis was established by studies on systemic vasculature. Thus, P2Y2 effects on the proliferation of PAECs and PASMCs have not yet been investigated.

### 5.5. Pulmonary Angiogenesis

The formation of new blood vessels in the cardio-pulmonary system from the preexisting vasculature plays a vital role in maintaining cardio-pulmonary vascularization and perfusion in various physiological and pathological conditions [105,166,167,168,169,170,171,172,173]. On the other hand, impaired angiogenesis has fatal consequences through a severe reduction or elevation of cardiac capillary density that contributes to dysfunction and heart failure [172,174,175,176]. P2Y2 has been suggested to affect endothelial cells, by promoting sprouting, angiogenesis, and vasculogenesis, and to protect the heart from ischemic damage [78,177].

There is experimental evidence supporting an important role of P2Y receptors in vasa vasorum neovascularization and pathologic vascular remodeling in PH [178]. Furthermore, in plexiform lesions from PAH patients, expression of CD39/ENTPD1, the ectonucleotidase responsible for the conversion of the nucleotides ATP and ADP to AMP, was decreased, suggesting that increased extracellular ATP level in the pulmonary vascular endothelium and within the angiomatoid proliferative lesions might contribute to excessive endothelial proliferation [44].

### 5.6. Blood Lung Barrier Dysfunction and Permeability of PAECs

In various experimental PH models, the initial increase in lung vascular leakage strongly correlated with the pulmonary pressure elevation [155]. P2Y2 was suggested to mediate the enhancement of adherent junctions and blood vessel permeability via ATP, Rac1, VEGFR-2, and VE-cadherin [110]. P2Y2, hence, significantly increased the trans-endothelial resistance in PAECs, which is of significant clinical relevance in terms of vascular permeability [46,48,179,180,181]. Tight junctions between endothelial cells are directly influenced, by increasing the gap formation through the actions of eNOS [179]. PAECs have not yet been sufficiently investigated concerning the stability of pulmonary endothelial tight junction formation and vascular permeability.

### 5.7. P2Y2 in Pulmonary Hypertension

The role of P2Y receptors in the pulmonary vasculature was investigated in several PH models. Significant upregulation of P2Y2 expression was revealed in the lungs of mice exposed to hypoxia [44]. Expression of P2Y2 along with other P2Y receptors, such as P2Y1, P2Y6, and P2Y11, was detected in PAECs isolated from PAH patients [49,50,51].

P2Y2 endogenous agonists such as ATP, ADP, and adenosine were claimed to be effective pulmonary vasodilators, possibly via P2Y2 receptors, in various animal PH models [182,183,184,185]. Likewise, ATP-MgCl2 infusion reduced mean pulmonary artery pressure and pulmonary vascular resistance in piglets with hypoxia-induced PH [186] and in children with PH secondary to congenital heart disease [187]. Furthermore, in pigs with acute hypoxia-induced PH, both P2Y1 inhibition with the selective antagonist MRS2500, and P2Y12R inhibition with cangrelor decreased pulmonary artery pressure [50].

## 6. Pharmacological Importance of P2Y2 as a Potential PH Therapy

The natural endogenous ligands for P2Y receptors (Table 2) are the extracellular purine and pyrimidine mononucleotides, such as ATP and UTP [68,188,189,190], and dinucleotides, such as diadenosine polyphosphates [189,190,191]. These nucleotides are normally located in the cytosol. Under certain conditions, such as cellular damage or apoptosis, these nucleotides can be released outside of the cellular compartment and, thus, bind to P2Y receptors. The latter leads to the argument that P2Y receptors and their natural ligands trigger clearance signaling [192].

### 6.1. P2Y2 Agonists

The natural endogenous ligands for P2Y2 receptors, as mentioned before, are ATP/UTP and other less potent oligonucleotides (Table 2) [133]. Synthetic selective P2Y2 agonists are difficult to generate due to the similarities of the sequences between P2Y receptors [193,194]. MRS2768 is the only selective P2Y2 agonist that does not show any interactions with other human P2Y receptors, such as P2Y4 or P2Y6 [133]. The best-known P2Y2 selective agonist “MRS2768” has an EC50 = 1.89 µM [133,195]. Another available drug, PSB1114, is a potent P2Y2 agonist that displays selectivity for P2Y4 and P2Y6 receptors, where the concentration is increased up to 60-fold over EC_50_ [194]. Although Diquafosol, also known as Diquas, is considered a selective agonist that activates P2Y2, it shows affinity to the P2Y4 receptor [193]. Diquafosol was approved for use in Japan in 2010 to treat dry eye disease [123,193]. The P2Y2 affinity order decreases from UTP = ATP > ATP-gamma-S >> 2-methylthio-ATP = ADP [196].

Evaluations of studies that involve vascular and pulmonary P2Y2 and its ligands are of pharmacological importance. Targeting the ocular P2Y2 with INS365 (Diquafosol eye drop) in a rat dry eye model promoted aqueous tear secretion in animals that underwent surgical excision of their lacrimal glands [180]. Similarly, in vivo studies have also demonstrated that P2Y2 agonists UTP and ATP increased tear secretion in rabbit [197,198] and regulated ocular mucin production in isolated rabbit and human conjunctiva [199]. Activation of P2Y2/4 receptors with INS45973, a UTP analog, resulted in lower blood pressure via distinct mechanisms, involving potassium/calcium activated channel KCa3.1 and connexin Cx37 [112].

Additionally, the P2Y2 agonists UTP and diadenosine tetraphosphate mediated in vivo arteriogenesis in a murine model of hindlimb ischemia [177]. Denufosol and other P2Y2 agonists improved lung function in cystic fibrosis patients [35,36,37,200,201,202,203]. Interestingly, very few studies have shown a protective effect of the specific P2Y2 agonist MRS2768 on hypoxic cardiomyocytes [78]. Thus, treatment with MRS2768 enhanced stability and protected the heart from ischemic damage in vitro and in vivo [78]. MRS2768 induced vasoconstriction of porcine pancreatic arteries via P2Y2, whereas ADP induced vasorelaxation [134].

### 6.2. P2Y2 Antagonists

A few inhibitors that target P2Y2 receptors are currently commercially available. AR-C118925XX was considered a selective and competitive P2Y2 receptor antagonist (Table 2) [204,205]. This antagonist did not display any interactions with the group of other receptors that interact with G_q_ at 10 µM concentrations, taking into consideration that the IC50 = 1 µM [195,206]. AR-C126313 and PSB-416 have been shown to selectively target P2Y2 [207,208].

## 7. Conclusions

P2Y2 is expressed, at least at mRNA levels, in all different layers of the pulmonary vasculature (adventitia, media, and intima). Deletion, overexpression, or pharmacological targeting of P2Y2 receptors leads to various biological organ and cell type specific alterations. Exposing P2Y2-expressing cells to mechanical stress, FSS, or hypoxia significantly increases the transcriptional expression of P2Y2, which, in turn, alters the physiological function. P2Y2 is known to play a crucial role in controlling systemic vascular tone, the proliferation and remodeling of vascular cells, chemotaxis sensing, airway clearance, and angiogenesis.

Despite the fact that more than 80% of newly developed medications partially or entirely target GPCRs and despite the presence of GPCR-based PAH therapies, including bosentan, macitentan, and iloprost, a sufficient GPCR-associated therapy for PAH has not yet been developed. The aim of our review was to shed light on the potential pathological involvement of the P2Y2 receptor in PAH development and the underlying mechanisms.

Investigation of P2Y2, as a mechanosensitive GPCR and a crucial player in many biological processes, in different pulmonary vascular cell-types will help identify rationales for administration of its ligands as a potentially novel pharmacological approach in PAH treatment. Studies of the application of selective P2Y2 (MRS2768) to activate mechanosensing pathways in vitro, ex vivo, and in vivo will increase our understanding of the involvement of P2Y2 in PAH pathomechanisms and offer new prospects and approaches for overcoming the disease.

## Figures and Tables

**Table 1:** Expression of P2Y2 receptors in the pulmonary vasculature.

Cell Type	Species	Detection Level	Ref.
PAECs	Human	mRNA	[47,48]
	Bovine	Agonist/activity	[52]
	Rabbits	mRNA and agonist/activity	[45]
LMVECs	Human	mRNA	[48,53]
PASMCs	Rat	Agonist/activity	[43]
PFs	Human	mRNA	[54]
	Mouse	mRNA	[55]

PAECs, pulmonary arterial endothelial cells; LMVECs, human microvascular endothelial cells from lung; PASMCs, pulmonary arterial smooth muscle cells; PFs, pulmonary fibroblasts.

**Table 2:** P2Y2 specific and non-specific ligands.

Ligand	Action	Target	Ref.
ATP	Agonist	P2Y1, P2Y11, P2Y12, P2Y2, P2Y4, P2Y6, P2Y13	[71,209,210,211]
UTP	Agonist	P2Y1, P2Y11, P2Y2, P2Y4, P2Y6	[204,211,212]
2-thioUTP	Agonist	P2Y2, P2Y4, P2Y6	[194,213,214,215,216,217]
4-thio-UTP	Agonist	P2Y2, P2Y4	[193]
UTPγS	Agonist	P2Y2	[209,218]
5BrUTP	Agonist	P2Y2, P2Y6	[209]
Ap4A	Agonist	P2Y2, P2Y4, P2Y12, P2Y13	[93,127,209,219,220]
PSB1114	Agonist	P2Y2, P2Y4, P2Y6	[194,212,221,222,223,224]
Denufosol (INS37217)	Agonist	P2Y2, P2Y4, P2Y6	[35,36,37,146,200,201,202,203]
Diquafosol (INS365)	Agonist	P2Y1, P2Y2, P2Y4, P2Y6	[34,98,122,123,124,225,226,227,228]
INS45973	Agonist	P2Y2, P2Y4	[146,229]
MRS2698	Agonist	P2Y2, P2Y4, P2Y6	[212,230]
MRS2768	Agonist	P2Y2	[78,133,134,135,136,137,164,229]
AR-C126313	Antagonist	P2Y2	[207,230]
Reactive blue-2	Antagonist	P2Y2, P2Y4, P2Y6, P2Y11, P2Y13	[127,135]
AR-C118925XX	Antagonist	P2Y2	[34,206]
PSB-416	Antagonist	P2Y2	[208]
Suramin	Antagonist	P2Y1, P2Y11, P2Y12, P2Y2, P2Y4, P2Y6, P2Y1, P2Y13	[6,7,44,98,127,135,164,206,231,232]

## Data Availability

Not applicable.
